# DeepION: A Deep Learning-Based Low-Dimensional Representation Model of Ion Images for Mass Spectrometry Imaging

**DOI:** 10.1021/acs.analchem.3c05002

**Published:** 2024-02-20

**Authors:** Lei Guo, Chengyi Xie, Rui Miao, Jingjing Xu, Xiangnan Xu, Jiacheng Fang, Xiaoxiao Wang, Wuping Liu, Xiangwen Liao, Jianing Wang, Jiyang Dong, Zongwei Cai

**Affiliations:** † Interdisciplinary Institute of Medical Engineering, Fuzhou University, Fuzhou 350108, China; ‡ State Key Laboratory of Environmental and Biological Analysis, Hong Kong Baptist University, Hong Kong SAR 999077, China; § Department of Chemistry, Hong Kong Baptist University, Hong Kong SAR 999077, China; ∥ Department of Electronic Science, National Institute for Data Science in Health and Medicine, Xiamen University, Xiamen 361005, China; ⊥ School of Business and Economics, Humboldt-Universitat zu Berlin, Berlin 10099, Germany; ∇ International Joint Research Center for Medical Metabolomics, Xiangya Hospital, Central South University, 87 Xiangya Road, Changsha 410008, China

## Abstract

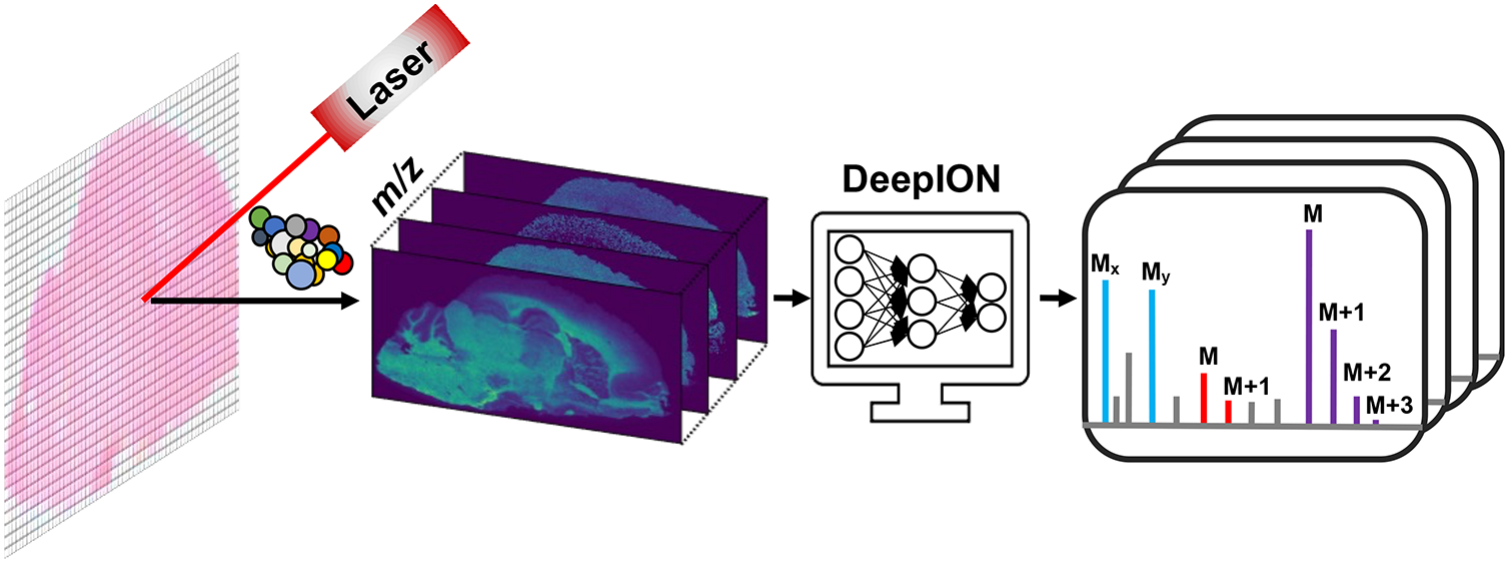

Mass spectrometry imaging (MSI) is a high-throughput imaging technique capable of the qualitative and quantitative in situ detection of thousands of ions in biological samples. Ion image representation is a technique that produces a low-dimensional vector embedded with significant spectral and spatial information on an ion image, which further facilitates the distance-based similarity measurement for the identification of colocalized ions. However, given the low signal-to-noise ratios inherent in MSI data coupled with the scarcity of annotated data sets, achieving an effective ion image representation for each ion image remains a challenge. In this study, we propose DeepION, a novel deep learning-based method designed specifically for ion image representation, which is applied to the identification of colocalized ions and isotope ions. In DeepION, contrastive learning is introduced to ensure that the model can generate the ion image representation in a self-supervised manner without manual annotation. Since data augmentation is a crucial step in contrastive learning, a unique data augmentation strategy is designed by considering the characteristics of MSI data, such as the Poisson distribution of ion abundance and a random pattern of missing values, to generate plentiful ion image pairs for DeepION model training. Experimental results of rat brain tissue MSI show that DeepION outperforms other methods for both colocalized ion and isotope ion identification, demonstrating the effectiveness of ion image representation. The proposed model could serve as a crucial tool in the biomarker discovery and drug development of the MSI technique.

## 1 Introduction

Mass spectrometry imaging (MSI) is a high-throughput molecular imaging technique that enables the spatial localization of thousands of biomolecules in tissue sections.^1^ The capabilities of MSI in biochemical characterization with spatial information promote its extensive applications in biology, clinical medicine, pharmaceutical research, and environmental science.^2−5^ When MSI acquires a full mass spectrum at each pixel of a tissue section, it produces thousands of ion images. Each image depicts the spatial distribution of specific ions or groups of ions. Co-localization refers to the quantification of the spatial correlation between ion images. When paired images from ions show high spatial similarity, they are termed colocalized ions. Identifying these colocalized ions is vital to interpreting complex MSI data in a biological context.

The identification of a colocalization ion involves looking up a similar spatial distribution ion by using distance-based similarity metrics. Although the high spectral resolution and rich spatial information in MSI makes them suitable for determining the subtle metabolic differences between regions of tissue, their high dimensionality, complex spatial structure, and low signal-to-noise ratios (SNR) also pose challenges in data interpretation, including similarity measurements of ion images. While image representation has been an important topic in computer vision and pattern recognition, it conveys information about the images by mapping raw image data directly into abstract semantic representations. Therefore, a “meaningful representation” of an ion image (an ion image representation) which captures significant spectral and spatial information to project onto a dense vector would help to reveal the intrinsic features embedded in the data and simplify the recognition of colocalized ions.^6^ Unlike the task of rendering ion images from MSI data, which is achieved by many commercial and home-built software packages,^7−9^ the ion image representation aims to mine hyperspectral MSI data for obtaining colocalized or isotope ion images which have similar spatial distributions.

Several methods have been developed to generate ion image representations. Supervised methods such as ColocML rely heavily on high-quality annotated data which makes them sensitive to the experiment artifacts and noise and reduces generalization capacities.^10^ On the contrary, unsupervised methods that abstract the underlying spatial patterns of individual 2D ion images without manual annotations are more practical in learning ion image representation.

Unsupervised methods for ion image representation can be roughly divided into three categories based on similarity measurement (SIM),^10−13^ dimensionality reduction (DR),^14,15^ and deep learning (DL)^16,17^ strategies, respectively. The SIM-based methods utilize vector-based distance calculations to quantify the spatial similarity of ion images through reshaping the original size of the image into a vector. DR-based methods first map the high-dimensional features into a low-dimensional embedding space and then measure the distance between the embeddings of ion images. Obviously, either SIM- or DR-based methods do not take the spatial information on pixels into account, which might be prone to missing relevant, localized differences between ions. Recent advances in deep learning frameworks for image representation in computer vision have opened up new opportunities for learning effective ion image representation.^18−23^ However, these DL-based methods are designed exclusively to handle the semantic relevant tasks of natural images, thereby being inadaptable for extracting image features of MSI data in the presence of plenty of noise and missing values. In addition, there is a special case in colocalization ions, i.e., isotope peaks natively from the same molecule (as shown in Figure S1). The ion images from isotope ions show not only the similarity in the spatial distribution but also the correlation of signal intensity. Therefore, discriminating the isotope ions from other colocalized ions of different molecules would assist the accurate and reliable molecular identification in MSI.

In this work, we propose DeepION, a new deep learning approach based on contrastive learning to generate effective ion image representations in a self-supervised manner without manual annotation. Two modes of DeepION, denoted as COL and ISO, are designed for the cases of regular colocalized ions from different molecules and isotope ions from the same molecule, respectively. As modality-specific data augmentation is critical to the performance of models trained by contrastive learning, we propose two data augmentation strategies especially for the COL and ISO mods by considering the characteristics of the Poisson distribution and missing patterns in MSI.^24^ The MSI data sets from two sections of rat brain tissues are used to assess the capability of DeepION on ion colocalization in comparison with SIM-based methods including the Euclidean distance, cosine distance, Pearson correlation coefficient (PCC), structure similarity index measure (SSIM),^25^ and determination coefficient R^2^, DR-based methods including principal component analysis (PCA), t-distribution stochastic neighbor embedding (t-SNE), and uniform manifold approximation and projection (UMAP), and other DL-based methods including ResNet18^26^ and SimSiam.^23^

## 2 Materials

### 2.1 Sample Preparation and Data Acquisition

Four-week-old SD rats are housed in sterile individually ventilated cages with a 12 h light/dark cycle at 22 °C and 45% relative humidity. The rats are sacrificed, and brain tissues are dissected and stored at −80 °C prior to section preparation. Experimental protocols are approved by the Hong Kong Baptist University Committee on the Use of Human and Animal Subjects in Teaching and Research.^27^

Rat brain tissue is cryosectioned at 10 μm using a CryoStar Nx70 cryostat (Thermal Fisher Scientific, Walldorf, Germany). The tissue cryosections are then transferred onto an indium tin oxide (ITO)-coated glass slide and placed in a vacuum chamber for half an hour before matrix application. Matrix solutions including 2,5-dihydroxybenzoic acid (DHB) and *N*-(1-naphthyl)ethylenediamine dihydrochloride (NEDC) are prepared at concentrations of 20 and 5 mg/mL in MeOH, respectively. A home-built pneumatic-assisted electrospray deposition system is used for matrix application at a flow rate of 15 μL/min for 13 cycles. MSI data are acquired at 50 μm spatial resolution using timsTOF fleX MALDI-2 (Bruker Daltonics, USA). A *m*/*z* range of 100 to 2000 was covered in both positive and negative ion modes. Raw MSI data exported from the instrument are about 4.4 GB (negative mode) and 4.7 GB (positive mode) in size.

### 2.2 Data Preprocessing

Data preprocessing, including peak picking, peak alignment, peak filtering, peak pooling, hotspot removal, and normalization, is conducted to generate ion images from raw MSI data. Here, peak picking and peak alignment are performed using SCiLs Lab software (Bruker Company, Germany). Peak filtering and peak pooling are performed using in-house Python scripts (details in our previous work^28,29^). After that, the raw MSI data is converted to a three-dimensional matrix *X*_(*M×N×H*)_, where *M* and *N* are the horizontal and vertical numbers of pixels, respectively, and *H* represents the dimensionality of ions corresponding to the *m*/*z* bins. For each ion image, those signals with intensities higher than 99% maximum intensity are termed hotspots and are truncated to eliminate the influence of extreme high intensities of ions on spectral normalization and visual inspection. Then the intensities of signals on an ion image are normalized to the range of [0, 1]. Taking the ion image *x* as an example, normalization is performed as follows:
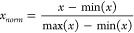
1

Finally, two MSI data sets containing 2165 images with a 192 × 425 shape (in negative mode) and 2263 images with a 198 × 422 shape (in positive mode) are obtained.

## 3 Methods

**Figure 1:**
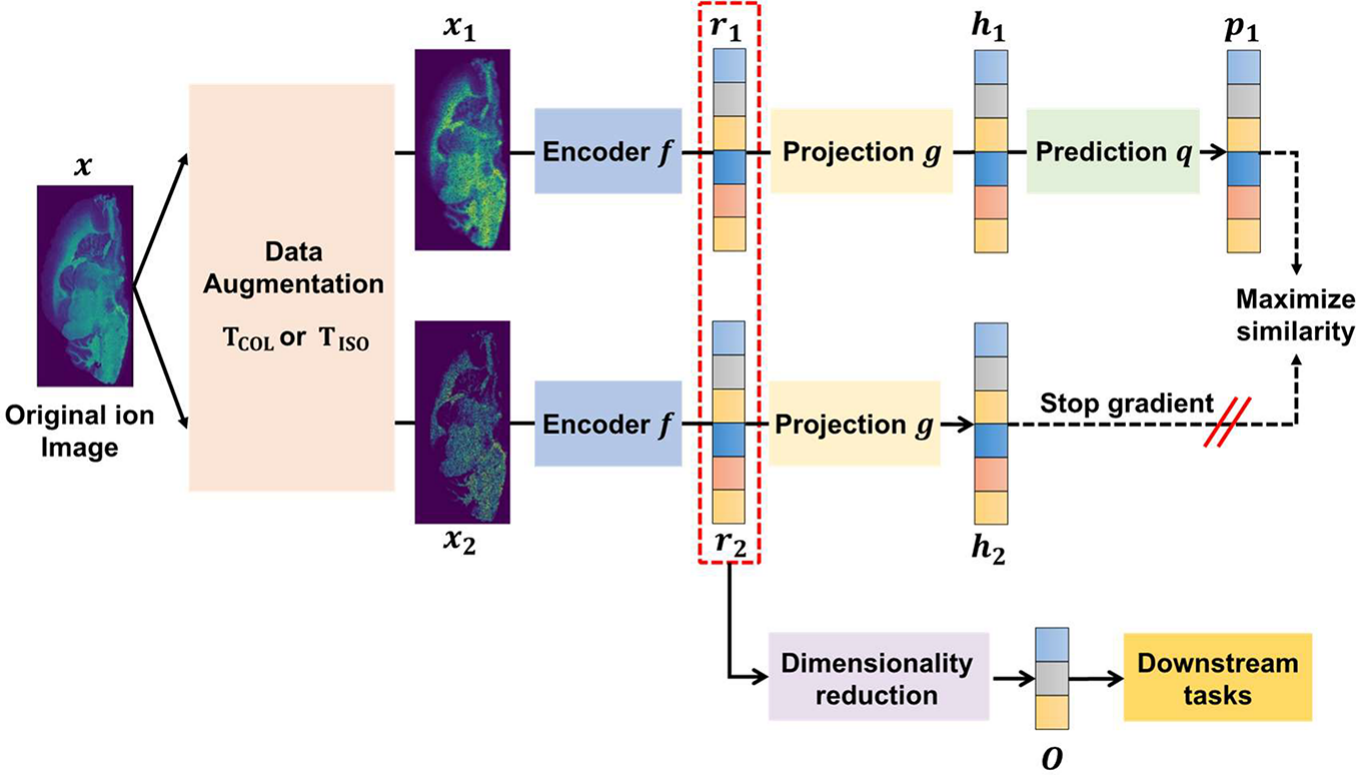
Schematic overview of DeepION consisting of four modules. (1) Data augmentation module. The original ion image *x* is first imported into the data augmentation module T to generate two augmented images *x*_1_ and *x*_2_, where the T_COL_, including color jitter, filtering, Poisson noise, and a random missing value, is carried in the COL mode, while T_ISO_ introduces an additional process for the intensity-dependent missing value in ISO mode. (2) Encoder module. Two images *x*_1_ and *x*_2_ are propagated through a pair of ResNet18-based encoders *f* that share parameters and then output two 512-dimensional representation vectors *r*_1_ and *r*_2_, respectively. (3) Projection module *g* and prediction module *q* are used to avoid collapsing solutions during the optimization process of maximizing the similarity between two augmentations from the same image^23^ and ensure the learning of the meaningful representation vectors *r*_1_ and *r*_2_. A contrasting loss is employed to maximize the similarity with a stop-gradient operation on *h*_2_ to prevent the reaction from collapsing during training. (4) Dimensionality reduction module. This module is applied to further reduce the dimensionality of ion image representation to a 20-dimensional vector *O* for downstream tasks.

The workflow of DeepION is shown in Figure 1. Two modes of DeepION, i.e., the COL and ISO modes, are designed for ordinary colocalized ions from different molecules and isotope ions from the same molecule, respectively.

### 3.1 Data Augmentation Based on MSI Prior Knowledge

**Figure 2:**
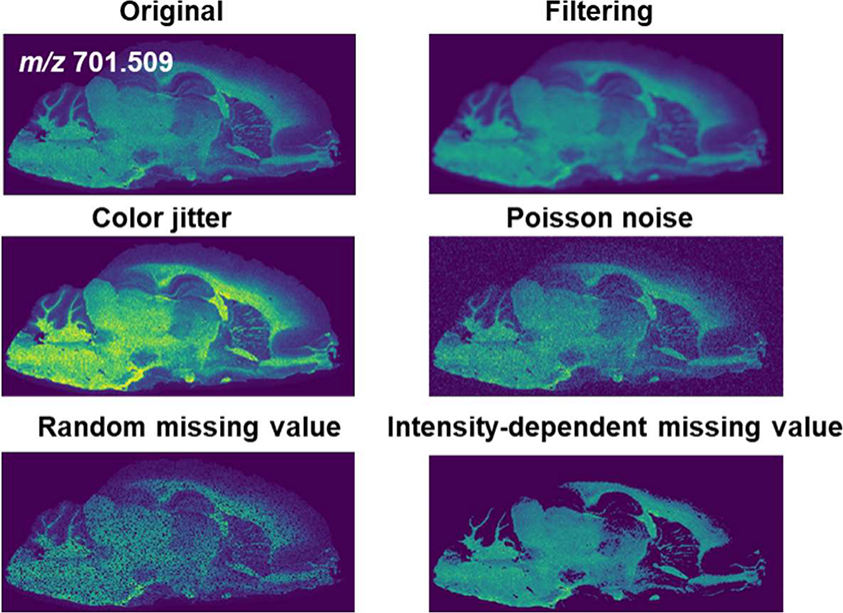
Designed data augmentation strategy.

Data augmentation, which involves applying different transformations to original images to produce new training data for the DeepION model, is crucial for contrastive learning.^18^ It is recognized that when the training data closely mirrors the distribution of the test data for a task, the model performs better. To ensure that the learned ion image representations are capable of abstracting high-level features of MSI, a modality-specific data augmentation strategy is designed by incorporating the characteristics of MSI data (detailed descriptions seen in Material S1), including color jitter, filtering, Poisson noise, random missing value, and intensity-dependent missing value (Figure 2). Here, color jitter and filtering are commonly used in natural images to make the model insensitive to the brightness of images and concentrate on the critical features. The operations of Poisson noise and random missing value are carried out in the COL mode of DeepION, which aims to simulate the data distributions within MSI, whereas the intensity-dependent missing value operation is conducted additively in ISO mode by setting the missing ratio of ions proportional to their intensities.

In practical applications, the COL mode and ISO mode of DeepION whose differences lay in the augmentation strategy are flexibly used for the identification of colocalized ions and isotope ions, respectively.

### 3.2 Architecture of the DeepION Model

The proposed DeepION model is constructed based on the SimSiam model,^18^ which aims to extract high-level spatial features from the ion image to obtain a meaningful ion image representation for each ion image in a self-supervised manner. DeepION is composed with the data augmentation module, encoder module, projection module, prediction module, and dimensionality reduction module.

The data augmentation module on the DeepION architecture is applied to create modified copies of the existing ion images with some minor changes, facilitating self-supervised training of the DeepION model.

The encoder module is used to learn an ion image representation function *f*(•|θ^*f*^) to extract the high-level features from ion images for downstream tasks. It consists of twin networks that share identical parameters and adopt the pretrained Resnet18 as the backbone network (Figure S2A). Taking the ion image *x* as an example, the encoder takes two augmented views *x*_1_ and *x*_2_ from ion image *x* as inputs. Then, two ion image representations *r*^1^ (*d* = 512) and *r*^2^ (*d* = 512) corresponding to the same image *x* are obtained as follows:
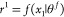
2
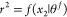
3

The target of projection is to learn a multilayer perceptron (MLP) function *g*(•|θ^*g*^) to ensure that the econder module outputs a meaningful ion image representation. Here, the projection module consists of three fully connected (FC) layers (as shown in Figure S2B). Then, two outputs *h*_1_ and *h*_2_ are obtained as follows:
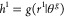
4
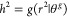
5

The target of prediction is to learn an MLP function to avoid the model producing the collapse results. It consists of two FC layers (as shown in Figure S2C). Then, outputs *p*_1_ and *p*_2_ are obtained as follows:
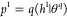
6
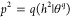
7

The target of dimensionality reduction is to generate a dense vector for similarity measurement and alleviate the adverse impact of the “curse of dimensionality” when the high-dimensional ion image representation *r* (*d* = 512) from the encoder is directly applied to the calculation. Since the UMAP algorithm has been demonstrated to perform better than other dimensionality reduction methods in many fields, we adopt it to obtain the denser ion image representation *O* as follows
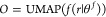
8where the reduced dimension is set to 20 by a trade-off between space–time complexity and information utilization. Then, to facilitate the calculation of similarity, a min–max scaler is adopted to adjust the range of features to [0, 1], as follows:
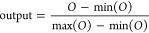
9

The output of the dimensionality reduction module is the low-dimensional ion image representation of an ion image that can be applied to downstream tasks.

### 3.3 Model Training

The Adam optimizer is adopted to train the encoder and prediction module, where the learning rate is set to 0.0003 and the momentum parameters β_1_ and β_2_ are set to 0.5 and 0.99, respectively. The initialization parameters of the DeepION model are set to follow the normal distribution *N*(0,0.02). Contrastive learning-based models often benefit from a large batch size so that the batch size is determined by the size allowed by the capacity of the GPU memory. The number of iterations is set to 100. Furthermore, to prevent the DeepION model from collapsing, we use the stop gradient operation during the model training. Specifically, the loss function of the DeepION model is as follows
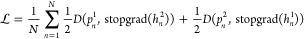
10where the encoder on *x*_2_ receives no gradient from *h*^2^ in the first term but receives gradients from *h*^2^ in the second term (and vice versa for *x*_1_).

### 3.4 Model Implementation

After model training, the output of the encoder is directly input into the dimensionality reduction module, and the output of the dimensionality reduction module is taken as the final output of the DeepION model. The DeepMSI model is implemented in Python using the PyTorch library, and the model is trained on a workstation equipped with a GPU Nvidia GTX 2080Ti graphics card.

## 4 Results and Discussion

The proposed DeepION is compared with SIM-based, DR-based, and the other DL-based methods for the task of identifying colocalized ions and isotope ions, respectively. The Euclidean distance is used to quantify the similarity between ion image representations generated by DR-based and DL-based methods. The performance of the algorithms is assessed by visual inspection and quantitative evaluations.

### 4.1 Colocalization Ion Searching

**Figure 3:**
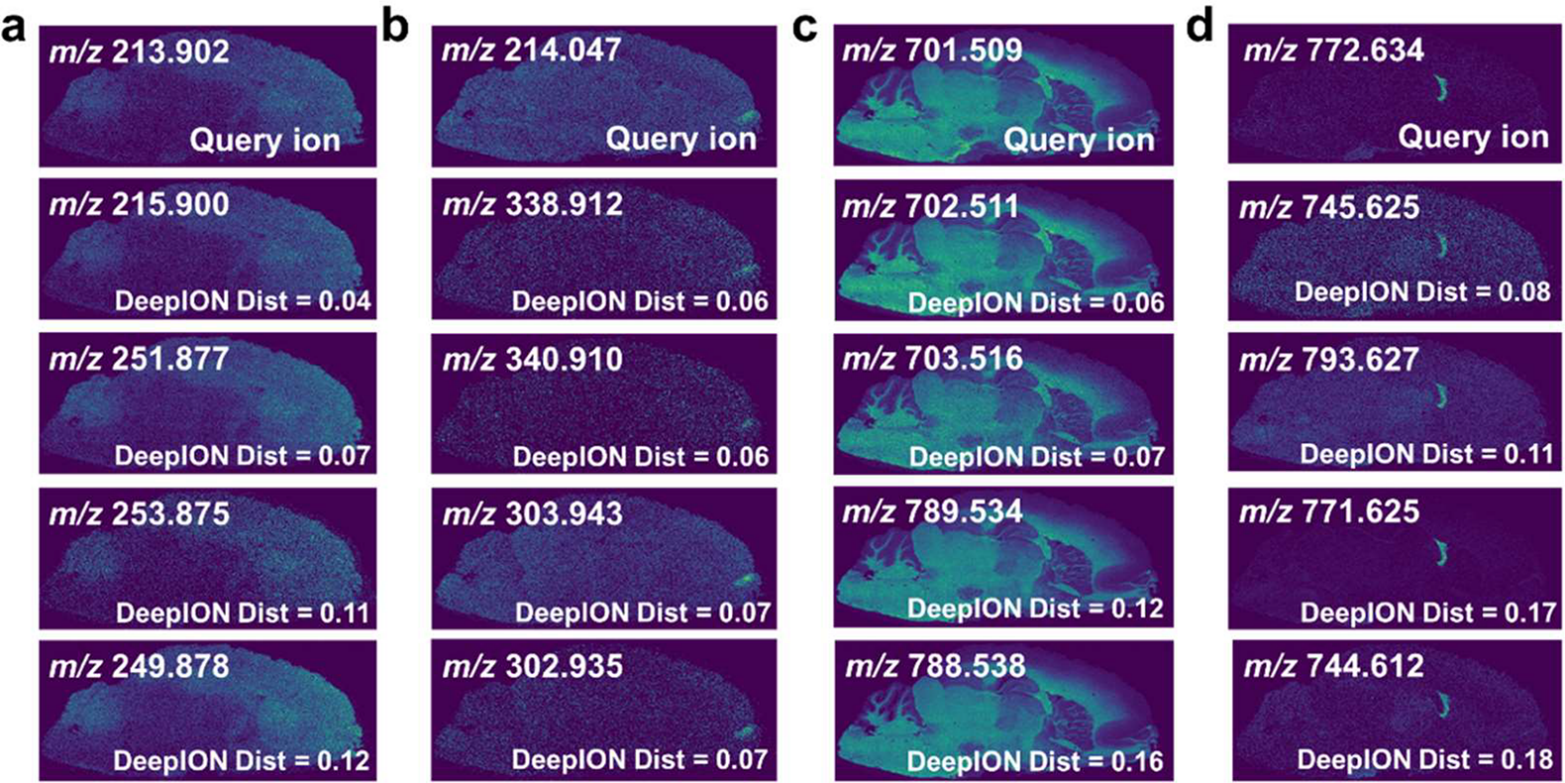
Identification of a colocalized ion for a representative query ion in negative mode. (a) *m*/*z* 213.902, (b) *m*/*z* 214.047, (c) *m*/*z* 701.509, and (d) *m*/*z* 772.634. A shorter distance indicates greater similar between the query ion and colocalized ion candidate.

Two data sets of MSI ion images are employed to investigate the capabilities of DeepION on colocalized ions. Here, we select five representative ions to be query ions to obtain the most similar ion images via calculating the distance between ion image representations (Figure 3, Figure S3). For negative ion mode, the high expression of query ion *m*/*z* 213.902 is observed on the locations of the cortex and cerebellum, while query ion *m*/*z* 214.047 is highly expressed in the olfactory bulb. Intuitively, the colocalization of ion pairs should preserve the structural similarities on tissue between their ion images. By using the DeepION model, the target ions *m*/*z* 215.900, *m*/*z* 251.877, *m*/*z* 253.875, and *m*/*z* 249.878 are recognized to be colocalized with *m*/*z* 213.902 (Figure 3a), while the target ions *m*/*z* 338.912, *m*/*z* 340.910, *m*/*z* 303.943, and *m*/*z* 302.935 are colocalized with *m*/*z* 214.047 (Figure 3b). The colocalization ions of query ion *m*/*z* 213.902 from R^2^, SSIM, PCA and UMAP (Figure S4) show partial consistency with DeepION by a visual inspection of the morphological structure of ion images. That is not the case for query ion *m*/*z* 214.047 (Figure S5), where misrecognized colocalized ions occurred with other methods. It is demonstrated that DeepION performs best in the task of discovering regular colocalized ions.

Then, a benchmark data set of ion images, which were manually annotated by experienced MSI technicians, is introduced for quantitative evaluation. Here, 98 ion images are manually divided into 17 colocalized ion categories within this data set, and details of the data distribution are shown in Figure S6. The performance of ion image representation is quantified by constructing a linear classifier to predict the label of each image in which leave-one-out validation is conducted to obtain the classification accuracy, similar to previous work.^18−20,23^ As shown in Table 1, ResNet18, SimSiam, and DeepION methods have higher accuracy than the other methods, which are attributed to the benefits of ion image representations from the inclusion of spatial information. In addition, the COL mode of the DeepION model achieves 86.73%, which outperforms the second-best SimSiam model by about 25%. The main difference between the DeepION model and SimSiam model lies in their data augmentation strategies. The SimSiam model uses a standard data augmentation designed for a natural image, while DeepION utilizes an MSI-specific scheme based on prior knowledge. These results demonstrate that the development of modality-specific data augmentation is critical to the performance of DeepION in the task of discovering colocalized ions.

**Table 1:** Comparison Results among Different Methods

Categories	Metric	ACC (%)
SIM-based method	Euclidean	12.24
	Cosine	31.63
	PCC	37.76
	R^2^	30.61
	SSIM	23.47
DR-based method	PCA	42.86
	t-SNE	14.29
	UMAP	40.82
DL-based method	ResNet18	52.04
	SimSiam	62.24
	DeepION	**86.73**

### 4.2 Isotope Ion Identification

**Figure 4:**
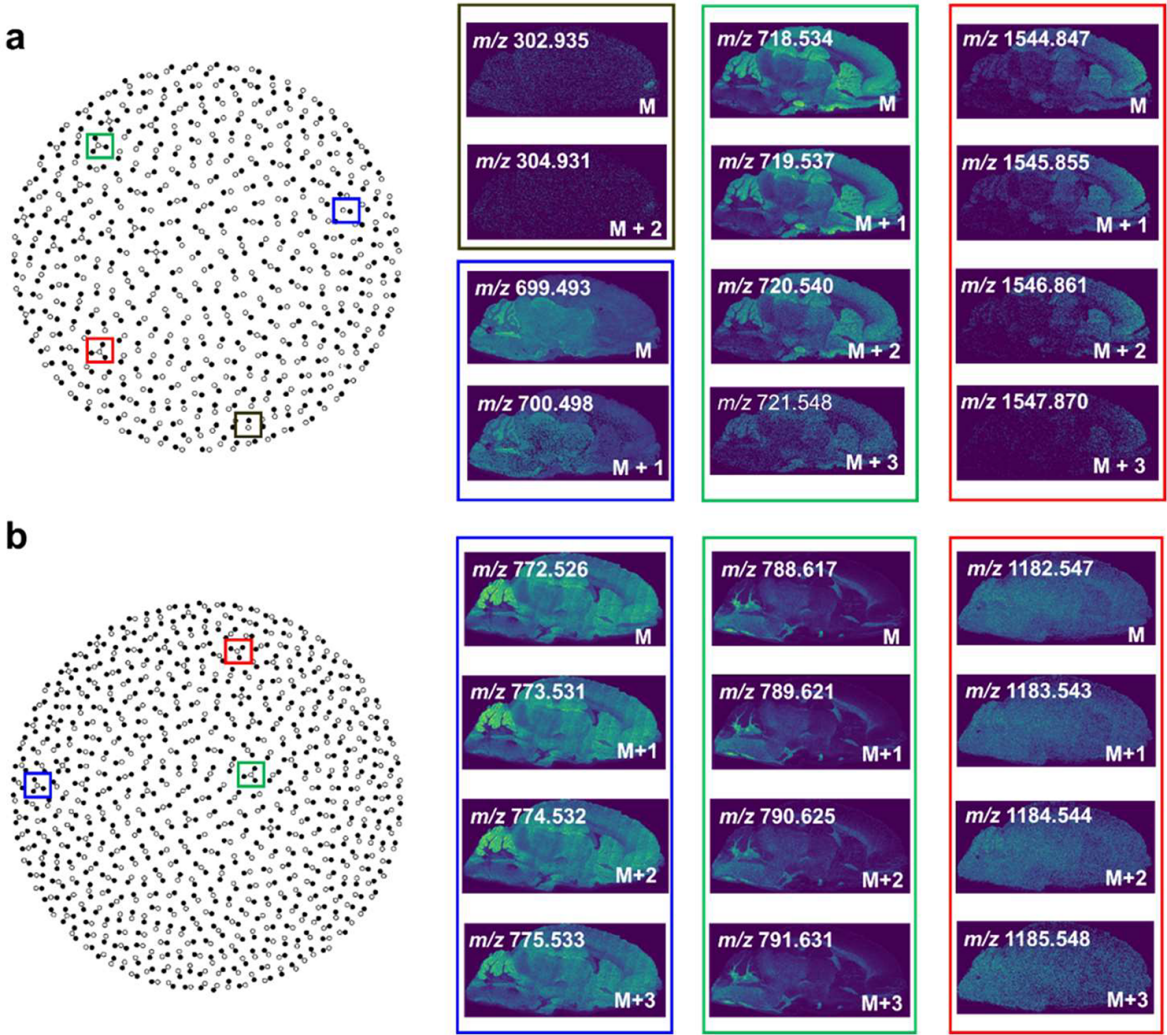
Isotope ion discovery for rat MSI data. (a) Negative ion mode. (b) Positive ion mode.

The ISO mode of the DeepION model is compared with two SIM-based methods, PCC and R^2^, which are used to identify isotope ions in MATASPACE^30^ and the rMSI package^31^ (details shown in Material S2). Figure 4 shows the colocalization results of monoisotopes (white node) and isotopes (black node) in which the edges represent the pair of colocalized monoisotope–isotope ions. Observing the results in the negative mode(Figure 4a), the relevant isotopes are accurately discovered for query monoisotpes *m*/*z* 302.935, *m*/*z* 699.493, *m*/*z* 718.534, and *m*/*z* 1544.847 using DeepION. Figure S7 and Table S1 show the colocalization isotope ions by PCC and R^2^. Although PCC and R^2^ could recognize the isotope for monisotopes with high SNR, they are both incapable of discovering the isotope *m*/*z* 1547.8702 for *m*/*z* 1544.8471 (PCC < 0.5, R^2^ < 0), where their proportions of missing values are 88.62 and 70.96%. Other results of isotope discovery are displayed in Figures S8 and S9. DeepION with ISO mode attains better performance for isotope discovery during visual inspection, which still detects the isotopes with low SNR.

In addition to visual inspection, we also conduct the quantitative evaluation for the ISO mode of DeepION. Here, monoisotope–isotopes are manually picked out and annotated one by one. The proposed DeepION model obtains 249 monoisotope–isotope pairs with 75.90% accuracy in negative mode and obtains 442 pairs with 92.76% accuracy in positive mode. The isotope ions identified by the ISO mode of DeepION are listed in Tables S2 and S3, where the rows colored in white and red indicate true and false discovery, respectively. The colocalized ions that are identified to have similar spatial distributions but do not belong to the isotope are colored in blue, for example, *m*/*z* 245.882 and *m*/*z* 247.879, or *m*/*z* 362.642 and *m*/*z* 364.639 (as see Figure S10). These probably happen when potential isotopic ions overlap with ions from the matrix or ions with similar *m*/*z* values that share identical spatial distributions.^32^ Additional chemical knowledge of molecules as a constraint of the model may further improve the performance of DeepION and facilitate more reasonable results. The proposed DeepION model introduces MSI domain knowledge to data augmentation to learn the characteristics of MSI data, which further improves the performance of DL-based methods in the task of discovering isotope ions.

## 5 Conclusions

Effective ion image representation means that ions with similar spatial distributions are close together in the embedded space, whereas ions with different spatial distributions are far away. It can facilitate the identification of colocalized ions and isotope ions. In this study, we present DeepION, a new DL-based method for ion image representation. The current results show that the DeepION model outperforms other previous methods in tasks of identifying colocalized ions and isotope ions.

The benefits of DeepION can be attributed to the following factor: (1) By introducing contrastive learning, the DeepION model targets the extraction of high-level spatial features from ion images to obtain a concise image representation in a self-supervised manner without manual annotation. The training objective of the model is to aggregate the augmented embeddings of the same sample and push away the embeddings from different samples. Figures 3 and 4 demonstrate the excellent abilities of DeepION in discovering colocalized ions and isotope ions, respectively. (2) By designing a novel data augmentation based on MSI domain knowledge, the DeepION model can further improve the image representational ability on the embedding space. The comparisons between SimSiam and DeepION prove the key roles of designed data augmentation in learning image representations of high-level molecular distribution features. (3) DeepION can be flexibly applied in the discovery of colocalized ions and isotope ions by implementing the COL mode and ISO mode, respectively. Figure S1 indicates the fine difference between colocalized ions and isotope ions so that it is necessary to design specific modes for two tasks. The proposed DeepION with COL mode outputs a similarity score for both colocalized ions and isotope ions, while the ISO mode gets only a similarity score on isotope ions.

In summary, the present results show the great potential of DeepION in ion image representation. The proposed method can not only be used to identify the colocalized ions but also can be applicable to isotope ions. Furthermore, the DeepION model is expected to be extended to multiple hyperspectral chemical imaging modalities, such as Raman and infrared microscopy. DeepION would be a promising tool for metabolite identification, biomarker discovery, and even metabolic flux analysis in spatially resolved metabolomics.

## Data Availability

The source code of the DeepION model together with the data set for testing are available on a public repository (https://github.com/BioNet-XMU/DeepION).

## Supporting Information Available

Data augmentation based on MSI prior knowledge; details of discovering isotope ions; difference between monoisotope–isotope ions and colocalized ions; architecture of each module in DeepION; identification of a colocalized ion for a representative query ion in positive mode; colocalization ion discovery for query ions *m*/*z* 213.902 and *m*/*z* 214.047 using different methods; data distribution of each colocalized ion category in the manually annotated data set; average mass spectra of four randomly selected regions; isotope ions identified in negative and positive modes; two examples that correspond to the blue marks in Table S2; isotope ion discovery for ions *m*/*z* 302.935, *m*/*z* 699.493, *m*/*z* 718.534, and *m*/*z* 1544.847 using PCC and R^2^; and isotope ions identified by the ISO mode of DeepION in the rat brain data set under negative and positive ion modes (PDF)
