# Supplementary material for: DeepION: A Deep Learning-Based Low-Dimensional Representation Model of Ion Images for Mass Spectrometry Imaging
Source: Anal Chem. 2024 Feb 20;96(9):3829–36. doi: 10.1021/acs.analchem.3c05002 (PMC10918617; doi:10.1021/acs.analchem.3c05002)
Supplement: Supplementary file 1 — ac3c05002_si_001.pdf [file ac3c05002_si_001.pdf]

Supporting Information

DeepION: a deep learning-based low-dimensional representation model of ion images for mass spectrometry imaging

Lei Guo[a,#], Chengyi Xie[b,c,#], Rui Miao[d], Jingjing Xu[d], Xiangnan Xu[e], Jiacheng Fang[b], Xiaoxiao Wang[b], Wuping Liu[f], Xiangwen Liao[*,a], Jianing Wang[*,b], Jiyang Dong[*,d], Zongwei Cai[*,b,c]

[a] Interdisciplinary Institute of Medical Engineering, Fuzhou University, Fuzhou 350108, China

[b] State Key Laboratory of Environmental and Biological Analysis, Hong Kong Baptist University, Hong Kong SAR, China

[c] Department of Chemistry, Hong Kong Baptist University, Hong Kong SAR, China

[d] Department of Electronic Science, National Institute for Data Science in Health and Medicine, Xiamen University, Xiamen 361005, China

[e] School of Business and Economics, Humboldt-Universitat zu Berlin, Berlin 10099, Germany

[f] International Joint Research Center for Medical Metabolomics, Xiangya Hospital, Central South University, 87 Xiangya Road, Changsha 410008, China.

This supplementary file includes:

1. **Supplementary Materials**

Material S1. Data augmentation based on MSI prior knowledge.

Material S2. Details of discovering isotope ions

2. **Supplementary Figures**

Figure S1. Difference between monoisotope-isotope ions and co-localized ions.

Figure S2. Architecture of the each module.

Figure S3. The identification of co-localized ion for representative queried ion in positive mode.

Figure S4. Co-localization ion discovery for query ion *m/z* 213.902 using different methods.

Figure S5. Co-localization ion discovery for query ion *m/z* 214.047 using different methods.

Figure S6. Data distribution of each co-localized ion category in the manually annotated dataset.

Figure S7. The average mass spectra of four randomly selected regions.

Figure. S8. The isotope ions identified in negative mode.

Figure. S9. The isotope ions identified in positive mode.

Figure S10. Two examples that correspond to the blue marks in Table S2.

3. **Supplementary Tables**

Table S1. Isotope ion discovery for ion *m/z* 302.935, *m/z* 699.493, *m/z* 718.534 and *m/z* 1544.847 using PCC and R^2.

Table S2. The isotope ions identified by the ISO mode of DeepION in the rat brain dataset under negative ion mode.

Table S3. The isotope ions identified by the ISO mode of DeepION in the rat brain dataset under positive ion mode.

1. Supplementary Materials

Material S1. Data augmentation based on MSI prior knowledge

Data augmentation plays an important role in contrastive learning. The commonly used data augmentation schemes in natural image include random cropping, random scaling, random masking, color jittering, Sobel operator, Gaussian noise, image filtering and random rotating etc[1]. However, they are inapplicable to the MSI data as follows:

Firstly, all ions are expressed in an identical region, e.g. the whole tissue section in which the major differences between ions are existed only in a fraction of pixels. It implies that the differences between ion images are more subtle than those in the natural images. Therefore, the strategies of data augmentation on strong alterations of spatial patterns such as random scaling, random masking, Sobel operator and random rotating, which often produce the positive samples extremely different from the initial image, may result in the failure of model training in MSI.

Secondly, natural images assume that the image noise is subject to the Gaussian distribution, whereas the signals and noise from MSI instrument satisfy the Poisson distribution according to previous studies[2].

Thirdly, there exist plenty of missing values in MSI data that lead to some zero-intensity pixels in an ion image. Especially the case of isotope ions, the pixels with lower intensity in an ion image are more likely to be missing.

Therefore, considering the differences on data characteristics of MSI ion images to natural images, we design the modality-specific data augmentation scheme for MSI data, including color jittering, image filtering, Poisson noise, random missing and intensity-dependent missing, as shown in **Figure 2**.

Material S2. Details of discovering isotope ions

Here is the process of isotope ions annotation.

Step 1: All ions are ranked by their *m/z* in an ascending order, and an ion set $\mathbf{I} = \{I_i\}_{i=1}^{N}$ is obtained;

Step 2: Calculate Euclidean distance between pairs of the ion representational vectors to obtain the similarity matrix $S = (s_{ij})_{i,j=1}^{N}$, where s_{ij} is the similarity score between I_i and I_j;

Step 3: Take sequential I_i $(i = 1,2,\cdots,N)$ as a candidate of monoisotope ion M, and search its isotope ions $\mathrm{M} + \mathrm{k}\ (\mathrm{k} = 1,2,\ldots,4)$ from $I_j\ (j > i)$ according to the following three criteria:

(1) the variation on *m/z* between I_i and I_j is lower than 5 ppm of the *m/z* value of I_i; (2) $s_{ij} < \theta_s$, where the threshold is set to $\theta_s = 0.25$; (3) if the isotope ion $\mathrm{M} + \mathrm{k}$ is not found, no more search for the isotope $\mathrm{M} + \mathrm{k} + 1$.

Step 4: Record the monoisotope ion I_i and its isotope ions found in the above procedure and remove them from ion set **I**, then return to Step 3 until the ion set **I** comes to null.

Table. Natural isotope abundance of some elements on Earth

(https://en.wikipedia.org/wiki/Natural_abundance)

Isotope	nat.abundance (%)	Atomic mass	Isotope	nat.abundance (%)	Atomic mass
^{1}H	99.985	1.007825	^{29}Si	4.67	28.97649
^{2}H	0.015	2.0140	^{30}Si	3.10	29.97376
^{12}C	98.89	12 (by definition)	^{32}S	95.0	31.97207
^{13}C	1.11	13.00335	^{33}S	0.76	32.97146
^{14}N	99.64	14.00307	^{34}S	4.22	33.96786
^{15}N	0.36	15.00011	^{35}Cl	75.77	34.96885
^{16}O	99.76	15.99491	^{37}Cl	24.23	36.96590
^{17}O	0.04	16.99913	^{79}Br	50.69	78.9183
^{18}O	0.2	17.99916	^{81}Br	49.31	80.9163
^{28}Si	92.23	27.97693			

2. Supplementary figures

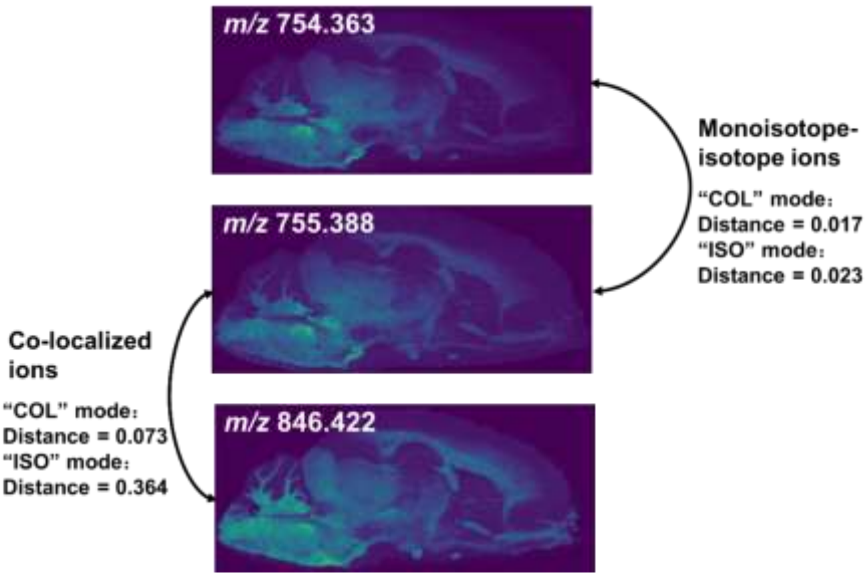

Figure S1. Difference between monoisotope-isotope ions and co-localized ions. Co-localized ions *m/z*755.388 and *m/z* 846.422 are both expressed in most areas of white matter, while monoisotope-isotope ions *m/z* 754.363 and *m/z* 755.388 not only expressed in most areas of white matter, but express higher in the white matter region of the cerebellum. The proposed DeepION with CO mode both gets similarity score on co-localized ions and isotope ions, while ISO mode only gets similarity score on isotope ions

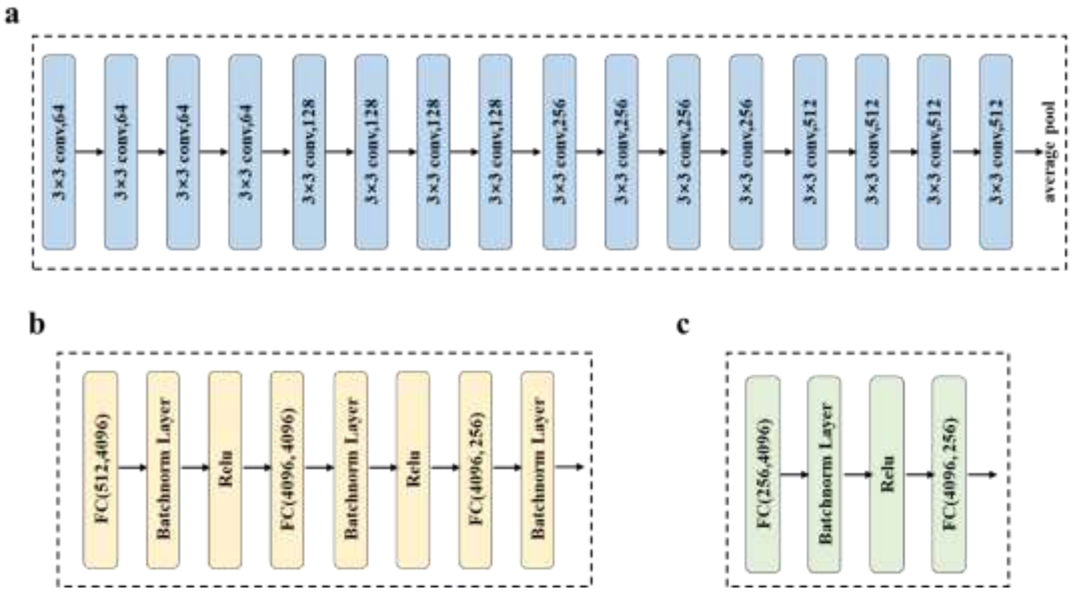

Figure S2. Architecture of the each module. (a) Encoder module; (b) Projection module; (C) Prediction module.

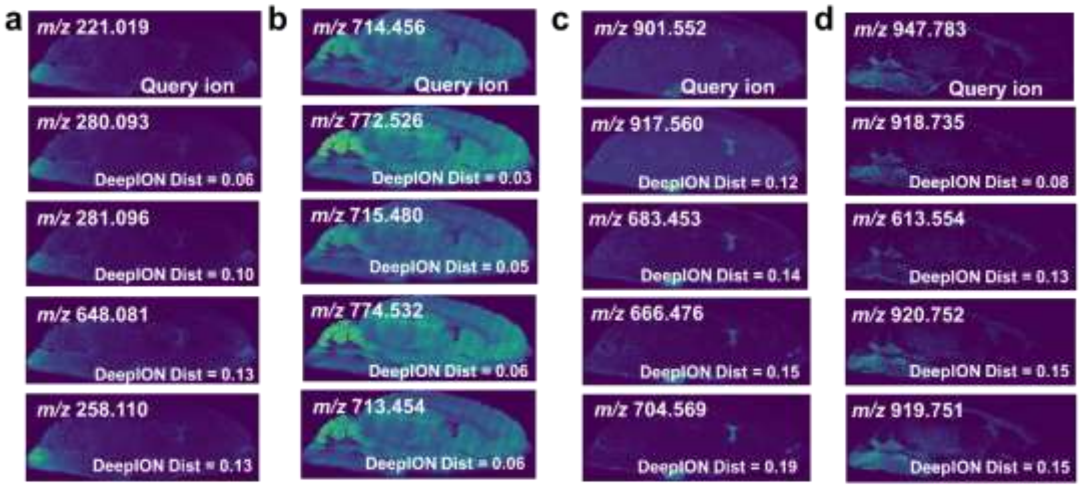

Figure S3. The identification of co-localized ion for representative queried ion in positive mode. (a) *m/z* 221.019; (b) *m/z* 714.456; (c) *m/z* 901.552; (d) *m/z* 947.783 in positive ion mode. The smaller distance means the more similar between query ion and candidate co-localized ion.

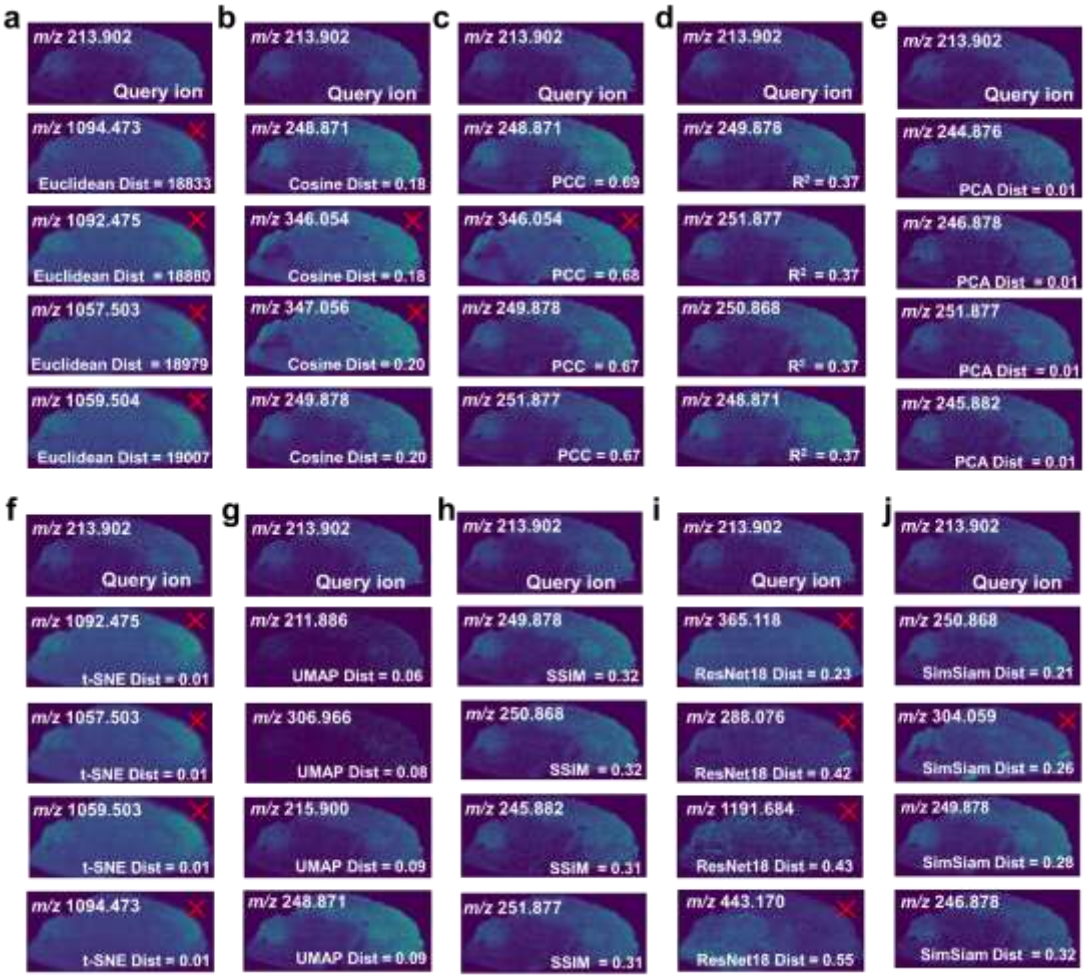

Figure S4. Co-localization ion discovery for query ion *m/z* 213.902 using different methods. The SIM-based methods (a) Euclidean distance; (b) Cosine distance; (c) PCC; (d) R^2; the DR-based methods (e) PCA; (f) t-SNE; (g) UMAP and the DL-based methods (h) SSIM; (i) ResNet18; (j) SimSiam are performed. Here, a smaller distance indicates a higher degree of similarity between the ion image and the query image. Conversely, for metrics such as PCC, R^2, and SSIM, a larger value means a closer resemblance between the two ion images. The error results of visual inspection are marked using red “×”

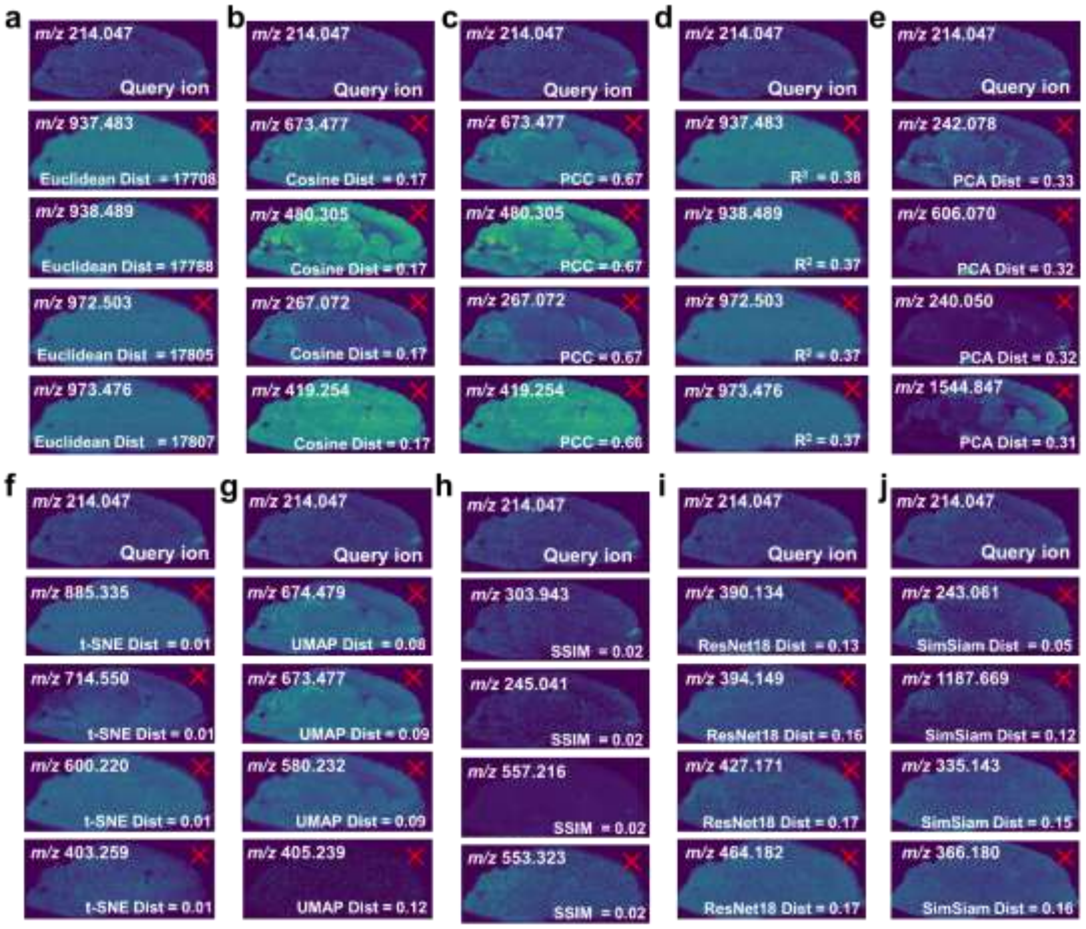

Figure S5. Co-localization ion discovery for query ion *m/z* 214.047 using different methods. The SIM-based methods (a) Euclidean distance; (b) Cosine distance; (c) PCC; (d) R^2, the DR-based methods (e) PCA; (f) t-SNE; (g) UMAP and the DL-based methods (h) SSIM; (i) ResNet18; (j) SimSiam are performed. Here, a smaller distance indicates a higher degree of similarity between the ion image and the query image. Conversely, for metrics such as PCC, R2, and SSIM, a larger value means a closer resemblance between the two ion images. The error results of visual inspection are marked using red “×”

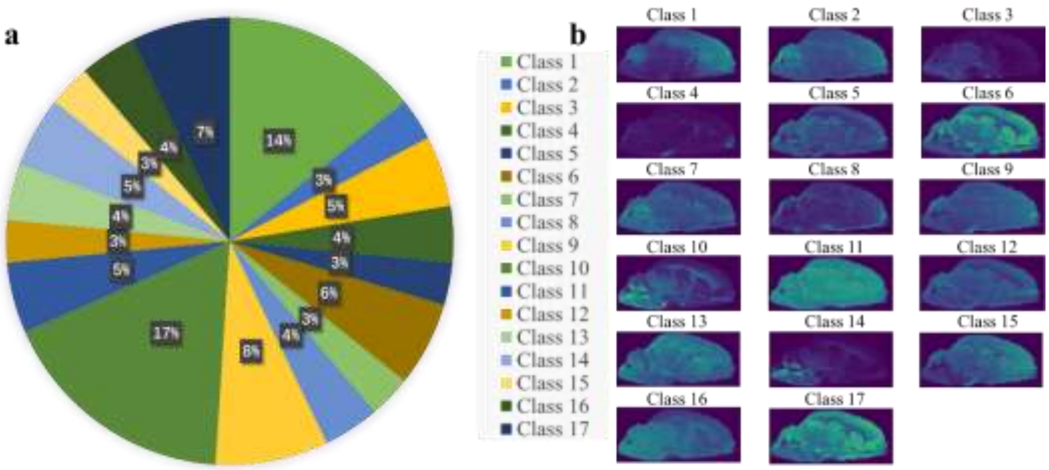

Figure S6. Data distribution of each co-localized ion category in the manually annotated dataset. (a) The proportion of the sample size for each class; (b) Average ion images obtained from each class sample in the dataset.

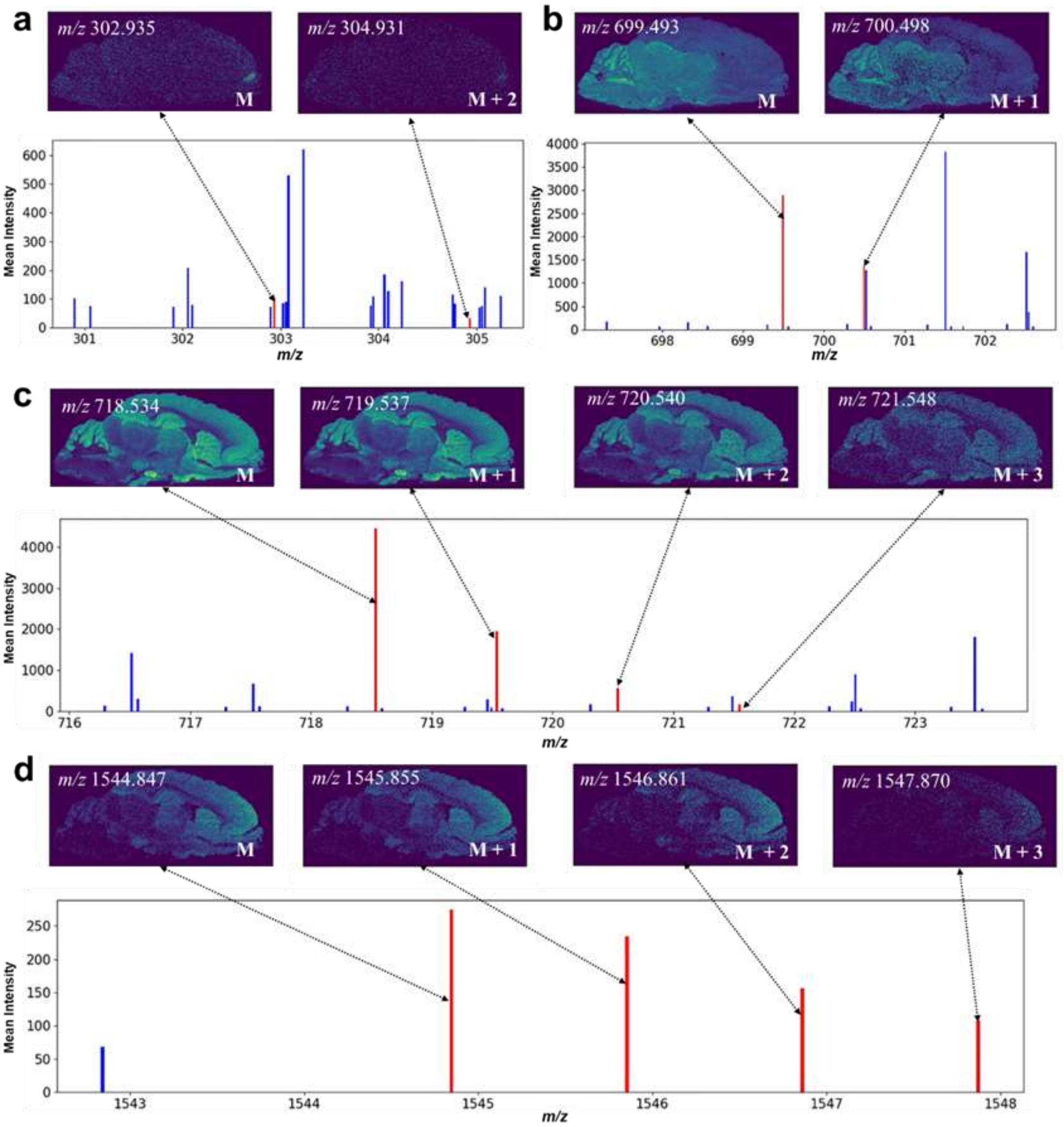

Figure S7. The average mass spectra of four randomly selected regions. (a) Monoisotope *m/z* 302.935 and isotope *m/z* 304.931; (b) Monoisotope *m/z* 699.493 and isotope *m/z* 700.498; (c) Monoistope *m/z* 718.534 and isotope *m/z* 719.537, *m/z* 720.540, 721.548; (d) Monoistope *m/z* 1544.847 and isotope *m/z* 1545.855, *m/z* 1546.861, *m/z* 1547.870.

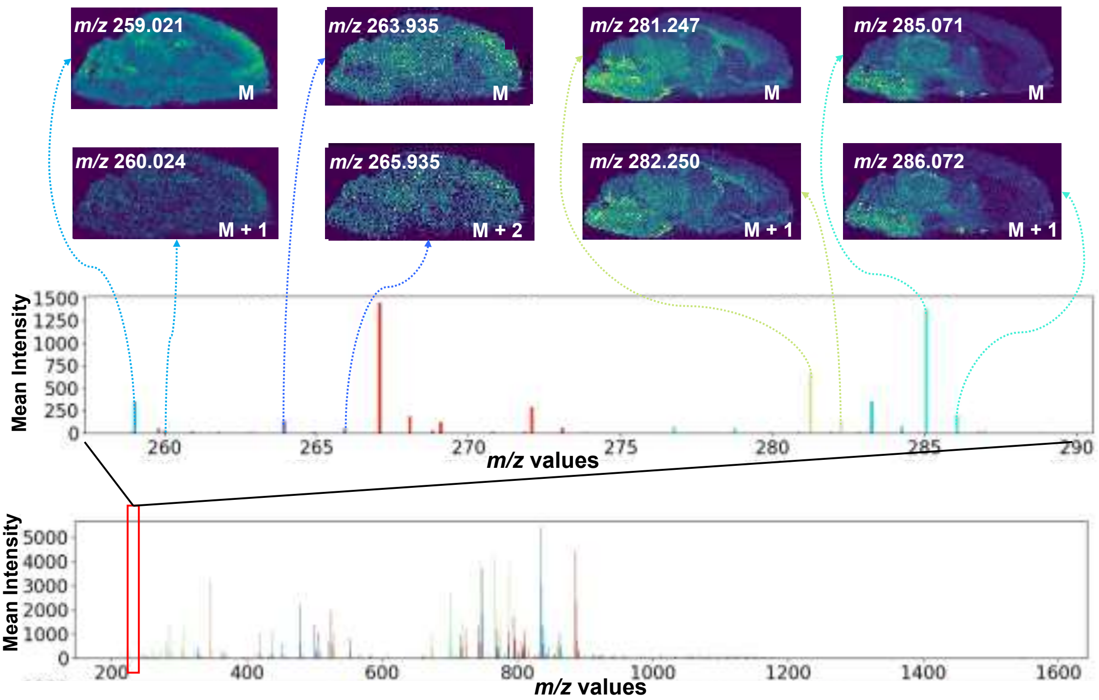

Figure. S8. The isotope ions identified in negative mode. The low m/z range are particularly displayed, ie. *m/z* 258 - *m/z* 290.

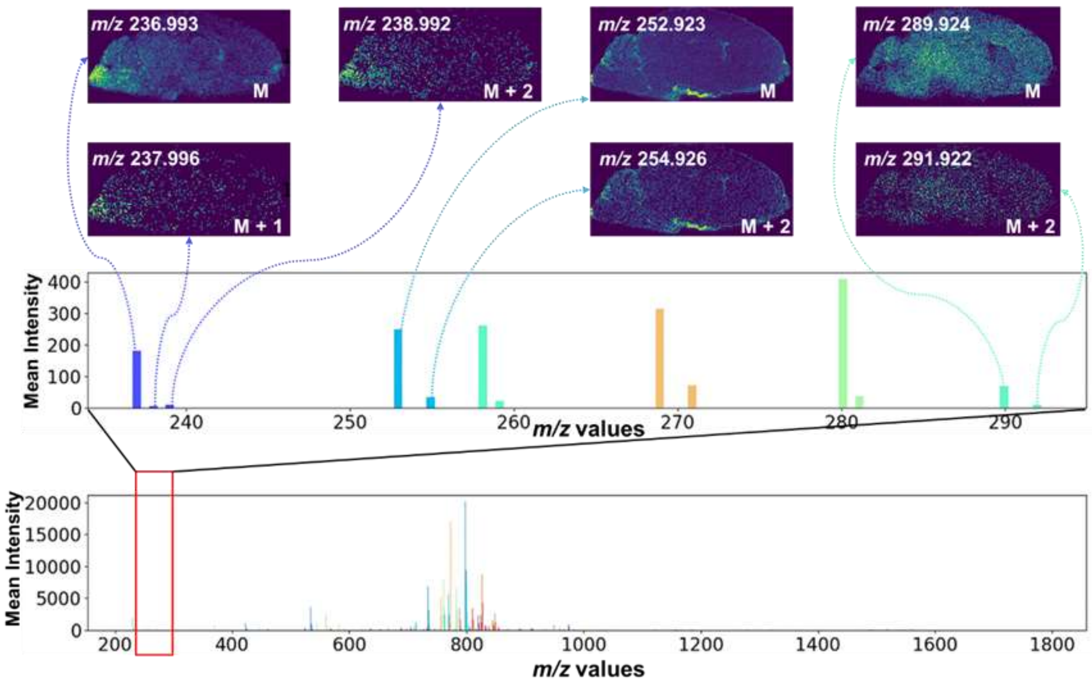

Figure. S9. The isotope ions identified in positive mode. The low m/z range are particularly displayed, ie. *m/z* 229 - *m/z* 298.

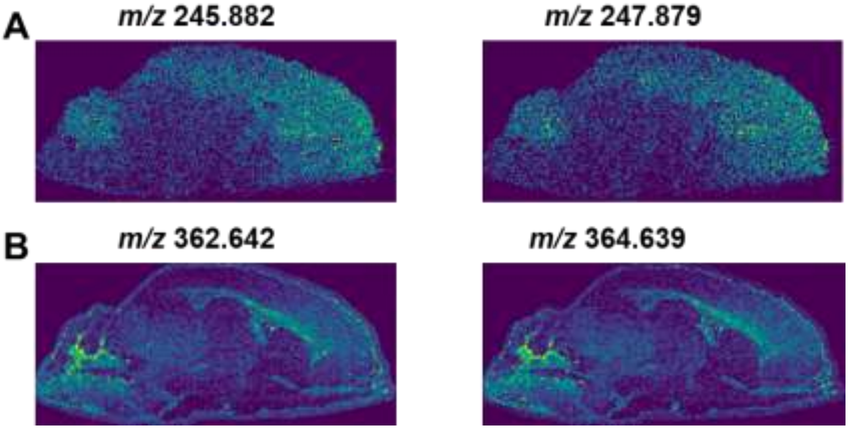

Figure S10. Two examples that correspond to the blue marks in Table S2.

Table S1. Isotope ion discovery for ion *m/z* 302.9345, *m/z* 699.4931, *m/z* 718.5343 and *m/z* 1544.8471 using PCC and R^2.

Monoisotope - isotope	PCC	R^2
m/z 302.934 – *m/z* 304.931	0.11	< 0
m/z 699.493 – *m/z* 700.498	0.82	0.12
m/z 718.534 – *m/z* 719.537	0.97	0.31
m/z 718.534 – *m/z* 720.540	0.92	< 0
m/z 718.534 – *m/z* 721.548	0.70	< 0
m/z 1544.847 – *m/z* 1545.855	0.79	0.61
m/z 1544.847 – *m/z* 1546.861	0.69	0.27
m/z 1544.847 – *m/z* 1547.870	0.42	< 0

Table S2. The isotope ions identified by the ISO mode of DeepION in the rat brain dataset under negative ion mode.

monoisotope (*m/z*)	isotope_1 (*m/z*)	isotope_2 (*m/z*)	isotope_3 (*m/z*)	isotope_4 (*m/z*)
**213.902**	**215.900**	---	---	---
219.945	221.944	---	---	---
221.833	222.836	**223.830**	---	---
226.034	227.037	---	---	---
240.050	241.052	---	---	---
242.078	243.082	---	---	---
243.061	244.063	---	---	---
244.081	245.085	---	---	---
**244.817**	**246.815**	248.807	---	---
**245.882**	**247.879**	---	---	---
**248.871**	**250.868**	252.865	---	---
**249.878**	**251.877**	---	---	---
258.933	260.932	---	---	---
259.021	260.024	---	---	---
259.803	261.804	---	---	---
260.901	262.898	---	---	---
263.935	264.937	265.935	---	---
**264.874**	**266.872**	---	---	---
267.072	268.074	**269.080**	**---**	---
268.802	270.797	---	---	---
269.953	271.951	---	---	---
271.852	273.852	---	---	---
272.087	273.089	---	---	---
**276.760**	**278.757**	---	---	---
281.247	282.250	---	---	---
283.242	284.245	---	---	---
283.263	284.266	---	---	---
285.071	286.072	---	---	---
286.773	288.768	---	---	---
286.976	288.973	---	---	---
290.085	292.080	---	---	---
298.067	299.072	---	---	---
**298.904**	**300.900**	---	---	---
302.935	304.931	---	---	---
303.231	304.235	---	---	---
303.943	305.940	---	---	---
**304.755**	**306.753**	---	---	---
306.075	307.078	---	---	---
309.278	310.281	---	---	---
320.751	322.745	---	---	---
323.026	324.029	---	---	---
326.766	328.763	---	---	---

327.231	328.234	---	---	---
331.262	332.265	---	---	---
331.938	333.938	---	---	---
336.151	337.155	---	---	---
336.719	338.717	340.707	---	---
346.054	347.056	---	---	---
348.915	350.913	---	---	---
362.048	363.051	364.056	---	---
362.236	363.239	---	---	---
**362.642**	**364.639**	---	---	---
366.638	368.636	---	---	---
369.205	370.209	---	---	---
**377.236**	**378.237**	---	---	---
**378.709**	**380.707**	---	---	---
384.726	386.722	---	---	---
**387.658**	**389.656**	---	---	---
392.975	393.979	---	---	---
395.067	396.068	---	---	---
402.739	404.735	---	---	---
**409.233**	**410.235**	---	---	---
411.049	413.045	---	---	---
**412.921**	**414.919**	---	---	---
**414.963**	**416.958**	---	---	---
417.239	418.243	---	---	---
419.254	420.257	---	---	---
**419.994**	**421.995**	---	---	---
421.264	422.267	---	---	---
422.951	424.952	---	---	---
**430.942**	**431.942**	**432.942**	---	---
**433.942**	**434.946**	---	---	---
435.248	436.252	---	---	---
435.948	437.947	---	---	---
436.944	438.942	---	---	---
437.264	438.267	439.270	---	---
451.943	452.945	453.942	---	---
452.275	453.278	454.273	---	---
457.232	458.235	---	---	---
462.064	463.067	---	---	---
468.254	469.260	---	---	---
**470.917**	**471.923**	**472.919**	**473.924**	---
**478.184**	**480.178**	---	---	---
478.291	479.294	---	---	---
480.305	481.309	482.311	483.315	---
485.263	486.264	---	---	---
500.275	501.278	---	---	---
**503.263**	**504.270**	---	---	---
506.286	507.289	---	---	---
506.321	507.325	---	---	---
**506.894**	**508.891**	510.888	---	---

508.335	509.339	510.343	---	---
508.569	510.565	---	---	---
515.913	516.915	517.914	---	---
524.275	525.279	---	---	---
524.295	525.298	---	---	---
528.269	529.273	---	---	---
528.306	529.308	---	---	---
540.051	541.054	542.061	---	---
552.270	553.274	---	---	---
565.044	566.046	---	---	---
**566.233**	**567.235**	---	---	---
599.316	600.320	---	---	---
**602.278**	**603.275**	---	---	---
602.505	603.512	---	---	---
606.070	607.076	608.081	---	---
**608.267**	**609.265**	---	---	---
645.502	646.505	---	---	---
**650.290**	**652.288**	---	---	---
659.503	660.503	---	---	---
**663.130**	**665.129**	---	---	---
664.619	665.623	---	---	---
**670.513**	**671.520**	---	---	---
673.477	674.479	675.488	---	---
**689.129**	**691.132**	---	---	---
**694.145**	**695.147**	---	---	---
695.964	697.965	---	---	---
**698.562**	**699.561**	---	---	---
699.493	700.498	---	---	---
701.509	702.511	703.516	---	---
715.569	716.568	717.576	---	---
716.518	717.522	---	---	---
718.534	719.537	720.540	721.548	---
723.493	724.496	---	---	---
**725.300**	**726.306**	---	---	---
726.589	727.587	---	---	---
**738.546**	**739.551**	---	---	---
742.534	743.539	---	---	---
744.549	745.555	---	---	---
746.560	747.563	---	---	---
747.494	748.496	---	---	---
752.602	753.598	---	---	---
754.614	755.616	---	---	---
756.584	758.582	---	---	---
762.560	763.556	---	---	---
762.607	763.611	---	---	---
**763.285**	**764.289**	---	---	---
764.518	765.519	---	---	---
766.533	767.538	---	---	---
768.535	769.540	770.526	---	---

**769.612**	**770.620**	---	---	---
770.565	771.569	---	---	---
771.625	772.634	---	---	---
772.580	773.585	774.588	---	---
**773.639**	**774.636**	---	---	---
774.537	775.541	---	---	---
**779.619**	**780.628**	---	---	---
780.399	781.401	---	---	---
**782.605**	**784.599**	---	---	---
784.041	786.039	---	---	---
786.524	787.526	---	---	---
788.538	789.534	---	---	---
790.534	791.538	792.546	793.547	---
**790.631**	**792.629**	---	---	---
794.552	795.556	796.559	---	---
797.414	798.423	---	---	---
800.443	801.445	---	---	---
802.425	803.421	---	---	---
806.534	807.537	---	---	---
806.608	807.609	---	---	---
808.543	809.548	---	---	---
808.623	809.625	810.635	---	---
810.524	811.526	---	---	---
**812.628**	**814.627**	---	---	---
816.393	817.394	---	---	---
818.594	819.597	---	---	---
**819.008**	**821.004**	---	---	---
826.670	827.673	---	---	---
830.434	831.435	---	---	---
834.523	835.525	836.533	**837.539**	---
834.592	835.599	---	---	---
836.623	837.623	838.635	839.632	---
838.553	839.558	---	---	---
842.415	843.419	---	---	---
845.422	847.428	---	---	---
846.422	848.429	---	---	---
851.439	852.440	853.430	---	---
**853.974**	**855.975**	---	---	---
854.437	855.433	---	---	---
857.513	858.514	---	---	---
860.633	861.634	---	---	---
862.645	863.646	864.651	865.654	---
864.398	865.403	---	---	---
866.430	867.431	---	---	---
874.440	875.448	---	---	---
876.438	877.444	---	---	---
883.526	884.532	---	---	---
885.547	886.546	887.551	888.557	---
886.642	887.637	---	---	---

890.404	891.413	---	---	---
890.685	891.685	---	---	---
**898.463**	**900.469**	---	---	---
906.672	907.668	---	---	---
911.472	912.475	---	---	---
**916.480**	**918.478**	---	---	---
924.475	925.478	926.485	---	---
**927.480**	**929.483**	---	---	---
931.486	932.487	---	---	---
933.488	934.487	935.486	---	---
943.481	944.476	945.473	---	---
955.484	956.495	---	---	---
957.490	958.501	---	---	---
959.494	960.494	---	---	---
**965.475**	**967.476**	---	---	---
975.483	977.488	---	---	---
1011.526	1012.533	1013.526	---	---
1032.526	1033.522	1034.531	---	---
**1042.535**	**1043.539**	**---**	---	---
1048.515	1050.517	---	---	---
1054.506	1056.501	---	---	---
1057.503	1058.505	1059.503	1060.513	---
1062.501	1063.495	1064.505	---	---
1069.499	1070.507	1071.501	---	---
**1081.510**	**1082.510**	---	---	---
1083.490	1084.499	**1085.497**	**1086.501**	**1087.493**
1092.474	1093.472	**1094.473**	**1095.475**	**1096.483**
1109.498	1110.494	---	---	---
1111.500	1112.501	1113.499	---	---
1114.497	1115.497	---	---	---
1118.482	1119.484	1120.483	---	---
1122.482	1123.490	---	---	---
1144.486	1145.482	---	---	---
1146.484	1147.488	1148.484	---	---
1151.482	1152.482	1153.473	---	---
1155.462	1156.468	1157.463	---	---
1158.560	1159.574	1160.575	---	---
1161.588	1162.590	---	---	---
1162.264	1163.276	---	---	---
**1163.587**	**1165.593**	---	---	---
1166.608	1167.603	---	---	---
1168.283	1169.288	**1170.274**	**1172.271**	---
1168.619	1170.613	1172.617	---	---
**1171.281**	**1173.276**	---	---	---
1173.617	1174.626	---	---	---
**1176.240**	**1177.243**	---	---	---
1176.628	1178.630	---	---	---
1178.223	1179.231	1180.228	1181.232	**1182.236**
1179.641	1180.641	1181.648	---	---

**1186.200**	**1188.207**	---	---	---
1194.234	1195.244	1196.242	---	---
1195.719	1196.721	---	---	---
1202.698	1203.700	---	---	---
1207.730	1208.733	1209.731	---	---
1211.734	1212.731	---	---	---
1213.750	1214.755	---	---	---
1253.758	1254.764	---	---	---
1289.739	1290.742	---	---	---
1544.847	1545.855	1546.861	1547.870	---
1572.882	1573.889	---	---	---

Table S3. The isotope ions identified by the ISO mode of DeepION in the rat brain dataset under positive ion mode.

monoisotope (*m/z*)	isotope_1 (*m/z*)	isotope_2 (*m/z*)	isotope_3 (*m/z*)	isotope_4 (*m/z*)
230.946	231.949	232.944	---	---
236.993	237.996	238.992	---	---
252.927	254.926	---	---	---
258.110	259.114	---	---	---
268.901	270.901	---	---	---
280.093	281.096	---	---	---
289.924	291.922	---	---	---
296.067	297.070	---	---	---
298.068	299.069	---	---	---
339.290	340.294	---	---	---
341.306	342.309	---	---	---
**369.352**	**370.356**	371.360	---	---
377.943	379.941	---	---	---
393.917	394.920	395.916	---	---
**414.891**	**415.898**	---	---	---
422.929	423.932	424.924	---	---
426.359	427.361	---	---	---
435.994	437.995	---	---	---
444.906	445.909	446.903	---	---
**459.877**	**460.881**	**461.882**	---	---
475.224	476.227	---	---	---
478.331	479.333	---	---	---
496.341	497.344	498.347	---	---
501.239	502.245	---	---	---
518.323	519.327	---	---	---
524.367	525.373	---	---	---
532.282	533.288	---	---	---
534.297	535.301	536.297	---	---
544.340	545.342	---	---	---
548.542	549.545	---	---	---
551.505	552.508	---	---	---
560.312	561.316	---	---	---
564.863	566.865	---	---	---
566.322	567.327	---	---	---
568.339	569.341	---	---	---
577.520	578.523	---	---	---
579.534	580.537	---	---	---
580.840	582.841	---	---	---
582.297	583.301	---	---	---
589.554	590.558	---	---	---
590.322	591.322	---	---	---
596.814	598.816	---	---	---
599.446	600.447	---	---	---

599.503	600.507	---	---	---
603.535	604.537	---	---	---
605.551	606.555	607.559	---	---
606.298	607.297	---	---	---
620.600	621.601	---	---	---
**622.301**	**623.305**	---	---	---
623.505	624.508	---	---	---
625.458	626.459	---	---	---
625.519	626.523	---	---	---
627.536	628.541	---	---	---
630.619	631.622	---	---	---
632.633	633.638	---	---	---
635.541	636.544	---	---	---
636.886	637.895	---	---	---
646.615	647.618	---	---	---
648.081	649.083	---	---	---
648.631	649.634	---	---	---
649.520	650.523	---	---	---
**651.118**	**652.118**	653.115	---	---
**651.535**	**652.539**	653.546	---	---
651.864	652.869	653.863	---	---
655.567	656.571	---	---	---
**660.377**	**662.371**	---	---	---
663.838	665.829	---	---	---
666.436	667.441	---	---	---
674.850	676.842	---	---	---
678.474	679.477	---	---	---
688.430	689.429	---	---	---
**689.496**	**690.503**	---	---	---
**694.468**	**695.467**	---	---	---
694.517	695.517	696.523	---	---
697.478	698.481	---	---	---
704.393	705.395	706.398	---	---
706.539	707.543	708.547	---	---
710.491	711.493	---	---	---
713.454	714.456	---	---	---
713.506	714.506	---	---	---
718.581	719.579	---	---	---
720.572	721.575	---	---	---
**726.465**	**727.471**	---	---	---
731.607	732.610	733.616	---	---
732.555	733.559	---	---	---
734.570	735.574	736.577	737.581	738.583
**734.633**	**735.631**	---	---	---
739.470	740.471	741.482	---	---
745.443	746.444	---	---	---
746.569	747.574	---	---	---
753.588	754.593	755.597	---	---
**754.536**	**755.544**	---	---	---

756.552	757.555	---	---	---
760.587	761.588	---	---	---
**760.652**	**761.653**	---	---	---
762.600	763.603	764.608	---	---
769.562	770.566	771.569	---	---
770.511	771.515	---	---	---
**770.618**	**771.619**	---	---	---
772.526	773.531	774.532	775.533	---
774.615	775.611	---	---	---
780.554	781.557	---	---	---
782.568	783.570	---	---	---
**782.638**	**783.641**	---	---	---
784.574	785.577	---	---	---
785.646	786.654	---	---	---
788.617	789.621	790.625	791.631	---
789.680	790.682	---	---	---
790.488	791.491	---	---	---
792.517	793.516	---	---	---
792.671	793.676	---	---	---
796.429	797.433	---	---	---
796.525	797.531	---	---	---
798.541	799.543	---	---	---
800.553	801.557	802.559	803.557	---
804.552	805.557	---	---	---
806.566	807.573	---	---	---
806.901	808.904	---	---	---
808.581	809.586	---	---	---
810.465	811.475	---	---	---
810.601	811.605	---	---	---
813.687	814.689	**815.697**	**816.701**	**817.704**
814.517	815.524	---	---	---
816.648	817.653	818.658	---	---
818.500	819.505	---	---	---
820.526	821.530	---	---	---
822.442	823.448	---	---	---
824.559	825.561	---	---	---
826.437	827.444	---	---	---
826.573	827.576	828.563	---	---
832.583	833.585	---	---	---
837.673	838.669	839.686	---	---
838.484	839.483	---	---	---
843.845	845.845	---	---	---
844.526	845.530	**846.536**	**847.538**	---
844.609	845.606	---	---	---
845.445	847.445	---	---	---
845.678	846.685	---	---	---
848.558	849.560	850.567	---	---
850.440	851.448	---	---	---
851.680	852.677	**853.677**	**855.667**	---

854.510	855.515	---	---	---
**859.698**	**860.696**	---	---	---
863.687	864.690	865.698	866.705	867.708
863.739	864.739	---	---	---
864.487	865.494	---	---	---
864.636	865.641	---	---	---
870.557	871.561	---	---	---
872.457	873.463	---	---	---
872.557	873.561	---	---	---
873.710	874.713	---	---	---
880.503	881.507	---	---	---
880.681	881.677	---	---	---
886.318	887.316	---	---	---
886.542	887.551	---	---	---
888.543	890.534	---	---	---
889.530	891.518	---	---	---
889.703	890.705	---	---	---
891.716	892.720	893.723	---	---
892.671	893.676	---	---	---
898.724	899.726	900.739	---	---
902.294	903.290	904.286	---	---
904.508	905.519	906.520	---	---
910.518	911.522	---	---	---
910.670	911.677	---	---	---
912.334	913.331	---	---	---
**912.575**	**913.582**	914.565	---	---
914.334	915.345	---	---	---
916.785	917.787	918.797	---	---
917.732	918.735	---	---	---
918.260	919.264	920.262	---	---
918.366	919.367	---	---	---
919.751	920.752	---	---	---
928.305	929.306	---	---	---
930.310	931.313	---	---	---
930.531	931.528	---	---	---
933.539	934.546	---	---	---
934.322	935.324	936.321	---	---
938.571	940.570	---	---	---
948.535	949.536	950.526	---	---
950.286	951.289	952.291	---	---
958.575	959.580	---	---	---
964.810	966.806	---	---	---
966.533	967.537	---	---	---
971.696	972.695	---	---	---
974.457	975.461	---	---	---
974.552	975.554	---	---	---
975.625	976.628	977.617	---	---
975.810	976.816	---	---	---
982.901	984.905	---	---	---

984.556	985.566	---	---	---
986.557	987.561	---	---	---
992.553	994.550	---	---	---
1002.481	1003.489	---	---	---
1002.581	1003.587	---	---	---
**1002.656**	**1003.654**	1004.651	---	---
**1005.898**	**1006.896**	---	---	---
1020.850	1022.858	---	---	---
1022.523	1023.528	---	---	---
1030.571	1031.571	---	---	---
1032.559	1033.559	1034.559	---	---
1036.827	1037.828	1038.828	---	---
1037.644	1038.641	---	---	---
1039.680	1041.681	---	---	---
1040.540	1041.548	1042.538	---	---
1045.544	1047.533	---	---	---
1053.600	1054.607	---	---	---
1055.651	1056.649	1057.651	---	---
1056.538	1057.537	1058.542	---	---
1059.652	1060.651	---	---	---
1060.497	1061.500	1062.501	---	---
1061.656	1062.660	---	---	---
1064.513	1066.519	---	---	---
1065.690	1066.690	---	---	---
1067.706	1068.710	---	---	---
1070.526	1072.527	---	---	---
1078.297	1079.300	---	---	---
1079.559	1081.561	---	---	---
1080.439	1081.439	1082.437	---	---
1083.677	1084.672	1085.665	---	---
1084.561	1085.573	---	---	---
1092.483	1093.488	---	---	---
1096.409	1097.413	---	---	---
1097.522	1098.516	---	---	---
**1100.550**	**1101.560**	---	---	---
1102.555	1103.559	---	---	---
1105.577	1106.576	1107.575	---	---
1110.519	1111.519	1112.523	---	---
1113.497	1114.494	---	---	---
1113.589	1114.594	---	---	---
1121.551	1122.555	1123.552	---	---
1123.417	1124.423	---	---	---
1124.541	1125.554	---	---	---
1126.503	1127.511	---	---	---
**1129.571**	**1130.565**	---	---	---
1134.532	1135.534	---	---	---
1137.540	1138.541	1139.543	---	---
1142.539	1143.542	---	---	---
1142.927	1143.936	1144.932	---	---

1143.404	1144.405	---	---	---
1146.545	1147.545	---	---	---
1150.556	1151.554	---	---	---
1156.534	1157.544	---	---	---
1158.899	1159.905	1160.905	---	---
**1161.541**	**1163.549**	---	---	---
1166.529	1167.533	1168.537	---	---
1169.513	1170.511	---	---	---
1172.517	1173.523	---	---	---
1174.879	1175.881	1176.890	---	---
**1176.531**	**1177.543**	---	---	---
1178.527	1179.532	---	---	---
1180.880	1181.887	1182.887	---	---
1182.546	1183.543	1184.544	1185.548	---
1188.522	1189.529	---	---	---
1189.669	1190.663	---	---	---
1190.859	1192.868	---	---	---
1195.528	1196.526	---	---	---
1196.858	1197.864	1198.868	---	---
1198.520	1199.524	---	---	---
1200.526	1202.527	---	---	---
1204.510	1205.516	1206.499	---	---
1205.640	1206.635	1207.640	---	---
1212.513	1213.514	---	---	---
1212.834	1213.835	1214.837	---	---
1214.503	1215.512	---	---	---
1215.685	1216.688	1217.689	---	---
1216.512	1218.518	---	---	---
1217.836	1219.844	---	---	---
1222.552	1223.551	1224.537	---	---
1225.538	1227.528	---	---	---
1228.812	1229.807	1230.810	---	---
1229.903	1230.906	1231.909	---	---
1231.663	1232.661	1233.666	1234.666	---
1232.522	1233.527	1234.524	---	---
1234.820	1235.817	1236.819	---	---
1237.512	1239.512	---	---	---
1240.507	1241.515	---	---	---
1242.509	1243.519	---	---	---
1243.708	1244.713	---	---	---
1247.635	1248.634	1249.636	---	---
1248.512	1250.505	---	---	---
1248.921	1249.924	---	---	---
1250.793	1251.788	1252.793	---	---
1251.885	1252.890	1253.896	1254.896	---
1255.919	1256.924	1257.930	1258.937	---
1257.540	1258.540	---	---	---
**1257.666**	**1258.673**	---	---	---
1259.686	1260.688	1261.681	---	---

1267.860	1268.874	1269.868	1270.865	---
1268.481	1269.496	---	---	---
1272.521	1273.527	---	---	---
1278.778	1279.779	---	---	---
1278.925	1279.923	1280.928	---	---
1279.533	1280.531	---	---	---
1281.925	1282.935	---	---	---
1287.520	1289.510	---	---	---
1291.898	1292.897	---	---	---
1292.538	1293.537	---	---	---
1293.753	1294.750	1295.760	---	---
1293.902	1294.902	1295.903	1296.901	1297.903
1294.530	1295.521	1296.541	---	---
1297.569	1299.559	---	---	---
1299.432	1300.430	---	---	---
1299.878	1300.885	---	---	---
1305.473	1306.472	---	---	---
1305.567	1306.563	---	---	---
1310.534	1311.534	---	---	---
1313.546	1314.541	1315.537	---	---
1316.534	1317.532	1318.530	---	---
1316.876	1317.874	---	---	---
1318.418	1320.424	---	---	---
1320.546	1321.546	---	---	---
1321.860	1322.859	---	---	---
**1323.861**	**1324.874**	---	---	---
1327.520	1328.526	---	---	---
1327.885	1328.891	---	---	---
1329.526	1330.524	---	---	---
1329.892	1330.900	---	---	---
1332.538	1333.541	1334.541	---	---
1332.881	1333.887	---	---	---
1334.918	1335.916	1336.905	---	---
1337.524	1339.515	---	---	---
**1339.860**	**1340.869**	---	---	---
1340.423	1342.432	---	---	---
**1341.871**	**1343.862**	---	---	---
1342.529	1344.517	---	---	---
1346.505	1347.509	---	---	---
1347.876	1348.881	---	---	---
1348.516	1349.525	1350.520	1351.520	---
1350.889	1351.885	1352.893	---	---
1355.896	1357.904	1359.875	---	---
**1356.533**	**1357.538**	**---**	---	---
1356.903	1358.893	---	---	---
1358.529	1359.528	1360.528	1361.531	---
1364.530	1365.533	---	---	---
1366.503	1367.501	---	---	---
1366.862	1367.857	1368.869	---	---

1369.872	1370.873	---	---	---
1370.513	1371.521	1372.519	---	---
1372.879	1373.878	1374.868	---	---
1374.521	1375.514	---	---	---
1375.882	1376.887	---	---	---
1376.525	1377.527	---	---	---
1377.896	1378.899	1379.895	---	---
1380.499	1381.507	1382.502	---	---
1383.851	1384.858	---	---	---
1384.515	1385.523	---	---	---
1386.528	1387.533	1388.536	---	---
1388.845	1389.845	1390.846	1391.855	---
1392.551	1393.551	1394.551	---	---
1393.875	1394.875	1395.876	---	---
1396.473	1397.483	1398.482	1399.482	---
1396.854	1397.846	---	---	---
1402.502	1403.508	---	---	---
1404.505	1405.510	---	---	---
1404.825	1405.822	1406.821	---	---
1406.504	1407.517	---	---	---
1408.521	1409.525	1410.526	---	---
1410.826	1411.828	1412.830	1413.823	---
1427.893	1428.893	---	---	---
1440.899	1441.905	---	---	---
1443.874	1444.871	1445.872	---	---
1453.908	1454.911	---	---	---
1466.150	1467.151	---	---	---
1468.149	1470.140	---	---	---
1469.897	1470.886	1471.894	1472.899	---
1479.919	1480.922	---	---	---
1481.929	1482.934	---	---	---
1485.862	1486.861	1487.865	---	---
1487.151	1489.134	---	---	---
1490.136	1491.153	1492.153	---	---
1494.172	1496.161	1498.167	---	---
1495.155	1497.167	---	---	---
1495.899	1496.902	---	---	---
1497.909	1498.913	---	---	---
1504.108	1505.112	---	---	---
1507.096	1508.095	1509.098	---	---
1507.187	1508.186	---	---	---
1508.506	1509.510	---	---	---
1513.167	1515.151	---	---	---
1513.886	1515.882	---	---	---
1516.132	1517.137	1518.146	---	---
1516.189	1517.194	---	---	---
1520.155	1521.163	---	---	---
1520.220	1521.223	---	---	---
1522.178	1523.183	1524.189	1525.191	---

1529.151	1531.132	---	---	---
1532.109	1533.109	1534.117	1535.130	1536.132
1534.520	1535.525	1536.527	---	---
1538.134	1539.146	---	---	---
1542.173	1543.176	---	---	---
1542.879	1543.890	---	---	---
1544.160	1545.160	---	---	---
1546.157	1547.166	---	---	---
1548.188	1549.198	1550.205	1551.209	1552.210
1554.090	1555.096	---	---	---
1558.159	1559.156	---	---	---
1560.135	1561.138	1562.133	1563.145	---
1564.146	1565.151	---	---	---
1566.138	1567.148	---	---	---
1568.865	1569.869	---	---	---
1570.176	1571.181	1572.183	1573.185	---
1576.224	1577.230	1578.231	---	---
1578.105	1579.100	---	---	---
1582.119	1583.120	---	---	---
1584.165	1585.171	---	---	---
1584.842	1585.841	1586.844	---	---
1586.150	1587.158	1588.150	1589.155	---
1590.148	1591.151	---	---	---
1592.153	1593.161	1594.169	1595.177	---
1596.191	1597.196	---	---	---
1598.207	1599.214	1600.214	1601.218	---
1604.107	1605.110	---	---	---
1606.122	1607.122	---	---	---
1606.825	1607.825	---	---	---
1608.131	1609.140	1610.142	1611.149	---
1612.180	1613.172	---	---	---
1614.172	1615.178	---	---	---
1616.171	1617.178	1618.168	1619.172	1620.188
1621.193	1622.203	---	---	---
1622.795	1623.801	---	---	---
1632.132	1633.141	---	---	---
1634.141	1635.151	---	---	---
1636.156	1637.163	---	---	---
1663.161	1664.159	---	---	---
1666.120	1667.124	---	---	---
1679.128	1681.111	---	---	---
1682.103	1683.100	1684.102	---	---
1692.150	1693.140	1694.139	---	---
1698.070	1699.072	---	---	---
1708.127	1709.118	1710.122	1711.123	1712.128
1718.153	1719.154	---	---	---
1720.160	1721.149	1722.148	---	---
1724.095	1725.091	1726.092	1727.106	---
1734.146	1735.139	---	---	---

1736.137	1737.144	1738.143	1739.143	---
1746.179	1747.183	1748.184	---	---
1750.121	1751.110	---	---	---
1752.117	1753.120	1754.120	---	---
1760.138	1761.146	---	---	---
1762.159	1763.164	1764.160	1765.162	---
1778.130	1779.139	1780.136	---	---

Reference

[1] Chen, X. L.; He, K. M. Exploring Simple Siamese Representation Learning. 2021 Ieee/Cvf Conference on Computer Vision and Pattern Recognition, Cvpr 2021 2021, 15745-15753. DOI: 10.1109/Cvpr46437.2021.01549.

[2] Verbeeck, N.; Caprioli, R. M.; van de Plas, R. Unsupervised machine learning for exploratory data analysis in imaging mass spectrometry. Mass Spectrom. Rev. 2020, 39 (3), 245-291. DOI: 10.1002/mas.21602.
